# Mucoepidermoid microcarcinoma in the context of parotid gland cyst

**DOI:** 10.1093/jscr/rjae230

**Published:** 2024-04-18

**Authors:** João P B Luizetti, Pedro P M Milanez, Mario A F Castro, Ricardo C de Almeida, Rogério A Dedivitis

**Affiliations:** Metropolitan University of Santos School of Medicine, Head and Neck Surgery Department, Av. Gal. Francisco Glycerio, 8 - Encruzilhada, Santos-SP, Brazil; Metropolitan University of Santos School of Medicine, Head and Neck Surgery Department, Av. Gal. Francisco Glycerio, 8 - Encruzilhada, Santos-SP, Brazil; Metropolitan University of Santos School of Medicine, Head and Neck Surgery Department, Av. Gal. Francisco Glycerio, 8 - Encruzilhada, Santos-SP, Brazil; Diagnos Medicina Especializada, Pathological Anatomy Department, Av. Siqueira Campos, 551, Santos-SP, Brazil; Metropolitan University of Santos School of Medicine, Head and Neck Surgery Department, Av. Gal. Francisco Glycerio, 8 - Encruzilhada, Santos-SP, Brazil; University of São Paulo School of Medicine, Head and Neck Surgery Department, Av. Dr. Arnaldo, 455 São Paulo-SP, Brazil

**Keywords:** mucoepidermoid carcinoma, parotid gland cyst, benign neoplasm of the major salivary gland

## Abstract

Mucoepidermoid carcinoma is a type of salivary gland cancer that can develop in the context of a parotid gland cyst. This type of tumor is composed of mucous, epidermoid, and intercalated cells, and usually presents as a slow-growing and painless mass. A parotid gland cyst is a condition in which a fluid-filled sac forms in the parotid gland. The tumor can be masked as it develops within the parotid cyst. A 45-year-old female patient presented with a suspect of benign neoplasm of the major salivary gland. She underwent partial right parotidectomy, which upon pathological analysis confirmed the diagnosis of mucoepidermoid microcarcinoma associated with parotid gland cysts. The patient did well and continues under regular follow-up with no further treatment.

## Introduction

Mucoepidermoid carcinoma (MEC) is the most common malignant tumor of the salivary glands, accounting for 5 to 10% of all tumors in this topography. Half of the cases affect the major salivary glands, mainly the parotid, and predominantly occur in females. This type of carcinoma receives its name due to its histological characteristics, where it presents three cell types: epidermoid, intermediate, and mucoid.

MEC is classified into degrees of malignancy (low, intermediate, and high). Low-grade tumors have higher proportions of mucoid cells and cystic areas. The cystic spaces are lined by mucoid cells, containing peripheral nuclei, which stain positive for PAS, with or without diastasis. Intermediate tumors present more solid areas, and high-grade lesions also show few mucoid cells. Additionally, they exhibit areas of pleomorphism, mitotic figures, perineural and bone invasion, as well as necrosis.

The diagnosis is usually clinical. The patient complains of painless, mobile, spherical, floating, and translucent swelling in front of the ear, whether left or right. In some cases, MEC can form within a parotid cyst, known as MEC intracystic. This process makes the cancer detection process difficult, as the cyst masks the presence of the tumor.

The objective of this study is to report a case of mucoepidermoid microcarcinoma in the context of a parotid gland cyst.

## Case report

**Figure 1:**
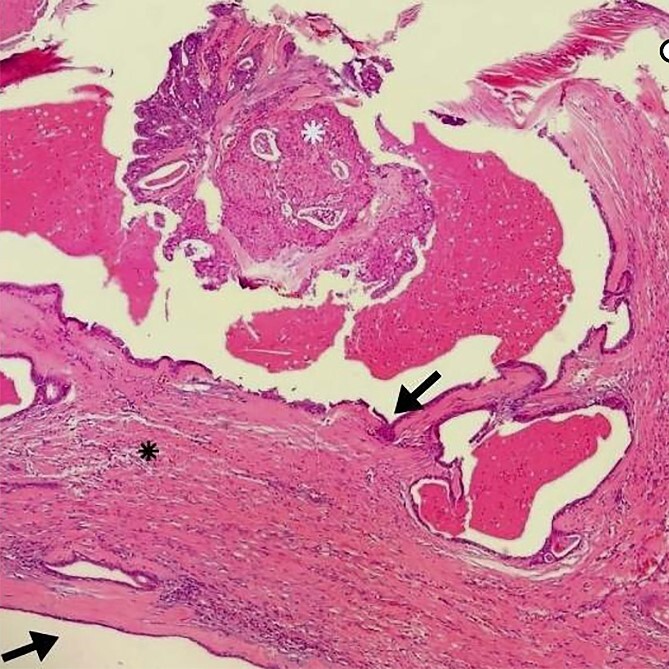
Arrows indicate the wall of a multilocular cyst without atypias. Black asterisk – non-invasive solid area with epidermoid alterations and low-grade atypias. White asterisk – invasive area with squamous and mucinous elements, suggesting the possibility of mucoepidermoid carcinoma. HE, ×4.

**Figure 2:**
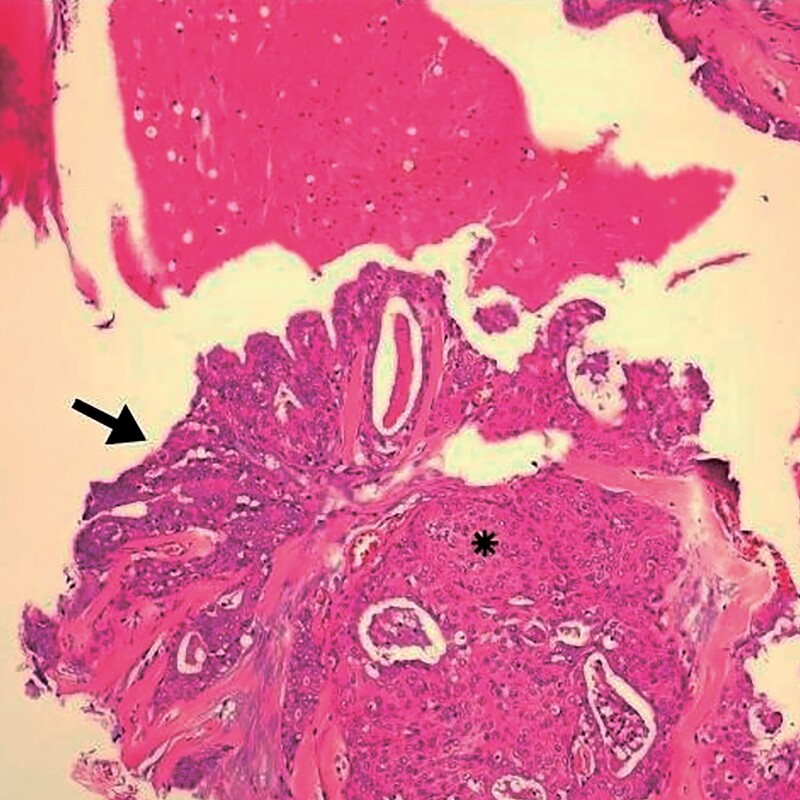
Detail of Fig. 1. Arrow indicates goblet cells and changes indicative of glandular epithelium. (^*^) area with squamous differentiation. HE, ×10.

A 45-year-old female patient, who denied smoking and alcohol consumption, experienced sudden discomfort in the right parotid region with an increase in volume for a week. The discomfort lasted two days and stopped after the prescription of non-hormonal anti-inflammatories. Although the discomfort passed, the volume continued to increase. The patient denied xerostomia, a history of rheumatoid arthritis, fever, and weight loss. On palpation, a firm, deep nodular lesion measuring 5 × 4 cm was detected, forming part of the right parotid gland. Ultrasonography confirmed the diagnosis of a right parotid cyst measuring 29 × 19 × 21 mm. Fine needle aspiration cytology (FNAC) was performed, with a diagnosis of chronic parotid cyst. As the lesion persisted and increased in volume, surgery was recommended; however, the patient chose to continue with observation. In subsequent examinations, the size of the lesion increased, measuring 6 × 4 cm. She underwent right partial parotidectomy with preservation of the facial nerve, with good evolution. In the context of the cystic lesion, a well-differentiated intracystic, well-defined mucoepidermoid microcarcinoma was diagnosed, with discrete cytological atypia (Figs 1 and 2). The patient remains under periodic follow-up, with no evidence of disease after six months.

## Discussion

In the case presented, the patient had a parotid cyst for several years, categorized as non-neoplastic. However, there was growth in the lesion, leading to the recommendation of surgery, but the patient chose observation. When she returned two years later, a 5 × 3 cm cyst was identified, integrated with the right parotid, and surgical resection with preservation of the facial nerve was again indicated. The histopathological result identified the presence of a well-differentiated intracystic low-grade MEC.

Mucoepidermoid carcinoma is known for its heterogeneity and histological variety. Its diagnosis is complicated by the lack of specific clinical characteristics; however, it presents itself as the most common malignant tumor of the parotid [1]. The size of the cyst is not directly related to the risk of malignancy. There is discussion about whether the formation of a MEC in a salivary gland cyst could be a process of evolution from a benign to a malignant cystic lesion [2].

Although fine-needle aspiration biopsy was performed before surgery, it may have limitations in detecting salivary gland tumors, as the sample obtained is small and may not be representative of the tumor as a whole [3].Fine-needle aspiration (FNA) generally does not provide sufficient information due to its cystic nature and insufficient sampling [4]. In practice, the cystic architecture of MEC can complicate the histopathological diagnosis.

The treatment of MEC is based on the surgical resection of the affected salivary gland. There is no indication for adjuvant treatment for early-stage tumors and low-grade differentiation. The discussion in this case revolved around the potential indication for total parotidectomy. However, considering it is an incidentaloma (millimetric tumor) with low grade and clear margins, a wait-and-see policy was opted for.

The presented case illustrates the importance of carefully evaluating parotid lesions, even when apparently benign, and highlights the possibility, albeit infrequent, of a parotid cyst evolving into a MEC. It is essential to be attentive to the clinical and radiological characteristics of parotid cysts and consider the possibility of malignancy, especially in cases of lesion growth or clinical suspicion [5].

## Conflict of interest statement

None declared.

## Funding

None declared.
